# Understanding HLA-DQ in renal transplantation: a mini-review

**DOI:** 10.3389/fimmu.2025.1525306

**Published:** 2025-02-05

**Authors:** Rajdeep Das, Neil S. Greenspan

**Affiliations:** University Hospitals Cleveland Medical Center, Case Western Reserve University School of Medicine, Cleveland, OH, United States

**Keywords:** renal transplantation, HLA-DQ, molecular mismatch, immunogenicity, risk stratification

## Abstract

Human leukocyte antigen (HLA) mismatching, particularly with HLA-DQ, significantly impacts the development of donor-specific antibodies (DSA) and transplant outcomes. HLA-DQ antibodies are highly immunogenic and detrimental, necessitating advanced high-resolution HLA typing to improve mismatch assessment and clinical risk evaluation. Traditional serological or low-resolution typing often misclassifies mismatches, leading to inaccuracies in assessing immunogenicity and predicting outcomes. Emerging molecular mismatch algorithms refine immunogenicity assessments by analyzing amino acid differences and structural interactions. These tools show promise for personalizing transplant protocols but have limitations, such as variability in predicting individual patient outcomes. Immunogenicity of mismatches also depends on evolutionary divergence and specific amino acid differences, with studies revealing that certain evolutionary lineages and polymorphisms influence T-cell alloreactivity and DSA development. Complexities in HLA-DQ protein expression, including combinatorial diversity of heterodimers and inter-isotypic heterodimers, further complicate risk evaluation. Expression levels, influenced by tissue specificity and inflammatory stimuli, and alternative splicing of HLA-DQ transcripts add additional layers of variability. Future clinical applications, enabled by high-resolution HLA typing, may include refined graft selection, improved DSA monitoring, and individualized therapy. However, understanding the precise mechanisms of HLA-DQ immunogenicity remains a priority for advancing transplantation science and enhancing patient outcomes.

## Introduction

Human leukocyte antigen (HLA) mismatches and the development of donorspecific antibodies (DSA) have long been recognized as crucial factors contributing to the success or failure of solid organ transplants (1, 2). Among the various HLA antigens, HLA-DQ has gained significant attention due to its strong association with transplant dysfunction and loss (3–5). Studies have highlighted that DSAs are more commonly formed against HLA-DQ compared to other HLA proteins, and these antibodies are often present in high titers and are particularly detrimental to transplant outcomes (6–8). This emerging understanding has spurred investigations into the immunogenicity of HLA-DQ, the pathogenic mechanisms underlying HLA-DQ antibodies, and the potential clinical applications of this knowledge to enhance transplant outcomes. This mini review focuses on HLA-DQ in kidney transplantation, but the fundamentalprinciples may also apply to other solid organ transplants, recognizing thatoutcomes can vary based on the specific organ involved. While we have made every effort to comprehensively address the topic, some aspects may still be left unaddressed, particularly given the scope of this being a mini review.

## HLA-DQ mismatching: typing methods and clinical implications

Accurate HLA typing is critical for defining HLA mismatches and understanding their clinical impact (9). Early research on HLA-DQ mismatching in solid organ transplantation relied on serological typing methods, which classified HLA-DQ antigens into broad categories such as DQ1, DQ2, DQ3, etc. However, recent advancements have allowed precise characterization of HLA-DQ providing a more detailed understanding of the immunogenic potential of these mismatches.

To achieve this level of detail, high-resolution HLA typing methods that accurately determine the amino acid sequences of HLA-DQ polypeptides are necessary. Unfortunately, much of the existing HLA-DQ typing data from solid organ transplants was obtained using low or intermediate resolution DNA-based methods or even older serological methods. When these lower resolution HLA-DQ types were compared with high-resolution two-field types determined by nucleotide sequencing, it was found that HLA-DQ mismatching assessed using the lower resolution typing methods was incorrect in 43% of donor-recipient pairs (10). This misclassification can have significant consequences, particularly in the assignment of DSAs, which in turn affects the observed relationships between HLA antibodies, long-term graft survival, and graft histology (11). Consequently, studies that utilized serological or low/intermediate level DNA-based HLA typing might provide misleading conclusions regarding HLA-DQ mismatching (12).

## Molecular mismatching and immunogenicity assessment

Recent research has focused on refining the assessment of HLA-DQ mismatches through molecular mismatching, which involves analyzing amino acid sequence differences using algorithms designed to predict immunogenic potential (13). Several such algorithms are under investigation, including HLA Matchmaker, EMMA (Epitope MisMatch Algorithm), PIRCHE-II (Predicted Indirectly Recognizable HLA Epitopes presented by HLA class II), and EMS-3D (ElectroStatic Mismatch Score).

HLA Matchmaker, introduced by Rene Duquesnoy, is a computational tool designed to analyze molecular mismatches in HLA (human leukocyte antigen) typing, primarily for transplant compatibility (14, 15). It remains widely used in the transplant community, focusing on comparing donor and recipient HLA alleles by assessing amino acid (AA) sequences. Originally based on “triplets” of AAs that are consecutive in the primary structure, the approach evolved to “eplets,” which group AAs at the molecular surface based on proximity in the tertiary as opposed to the primary structure. These eplets quantify mismatches, but their determination relies on assumptions regarding which mismatches should be considered eplets and which should not, and mismatches may be redundantly counted across multiple eplets on one antigen. To address the limitations of mismatch enumeration, alternative approaches have emerged.

The HLA Epitope MisMatch Algorithm (HLA-EMMA), developed by the Leiden group, introduces a new dimension by emphasizing the comparison of donor and recipient HLA class I and II AA sequences to identify polymorphic solvent-accessible mismatches likely to interact with B cell receptors, hypothesizing their immunogenic relevance (16). However, evidence supporting this concept is limited.

The ElectroStatic Mismatch Score (EMS-3D), developed by Vasilis Kosmoliaptsis, evaluates electrostatic potential at the protein surface on the assumption that this parameter will be useful to predict the likelihood of DSA generation (17). Although focused on a biophysical parameter instead of just AA disparity, its impact on immune activation remains uncertain.

Finally, the PIRCHE-II software, based on the Net-MHC algorithm, predicts HLA-derived T-cell epitopes that could be recognized through the indirect recognition pathway (18, 19). Despite ongoing refinement of the PIRCHE-II algorithm (20), it has shown a correlation with graft rejection and failure in various organ and donor types (21, 22). Additionally, PIRCHE-II scores for HLA Class II antigens are linked to an elevated risk of T-cell mediated rejection (TCMR) (23, 24). While PIRCHE-II could be a tool used in transplant immunology to predict immune responses to HLA mismatches, a potential limitation is in addressing pre-existing DSA. These pre-existing DSAs are crucial for assessing donor compatibility and the risk of rejection. However, the T-cell memory module of PIRCHE could help identify repeat mismatches from prior exposure to HLA antigens, providing additional insights into potential memory T-cell activation and immune risk (25).

Comparative studies of these molecular mismatching approaches for HLA-DQ have shown that eplet-based mismatching correlates more strongly with clinical outcomes than traditional antigen-level mismatching or simply counting amino acid differences (13, 26). Importantly, it is often more informative to calculate eplet mismatch scores for each individual HLA-DQ protein rather than as a cumulative score for all HLA-DQ mismatches (27). Molecular mismatch scores were shown to be useful for comparing HLA mismatches across different racial and ethnic populations and have been correlated with graft loss in multiple studies (28, 29). Based on these findings, some propose using molecular mismatching approaches for risk stratification of transplant patients, which include personalizing induction therapy, optimizing drug minimization protocols, refining post-transplant monitoring, and making informed decisions about donor selection (30). Despite these advances, the challenge remains to ensure that assessments of HLA-DQ mismatching are consistently correlated with the development of HLA-DQ antibodies and their pathogenicity. While there is intuitive and empirical support for the hypothesis that physicochemical differences correlate with immunogenicity, there is also concern that it may be premature to apply these tools in clinical practice, particularly for HLA-DQ. One of the concerns is while analyzing data across a large group of transplant patients, a statistical link might be observed between higher mismatch scores and increased rates of graft rejection or loss, but when looking at individual patients, the mismatch score alone may not accurately predict whether they will experience these negative outcomes (31). Essentially, there is a degree of variability within the patient population that cannot be fully captured by the mismatch score alone. Immunogenicity is a complex term that aims to describe and define the ability of the donor organ (in the specific case of transplantation) to provoke an immune response. It is indeed affected by the dissimilarity between donor and recipient, but probably not just by the degree of dissimilarity alone.

## Evolutionary divergence and functional differences in HLA-DQ

There is also evidence that evolutionary and functional divergence between donor and recipient HLA-DQ proteins influences immunogenicity. Some researchers have classified HLA-DQ mismatches into evolutionary groups based on the α chain, with one group consisting of heterodimers containing a DQα01 polypeptide (that include the serologic HLA-DQ1 alleles) and another group comprising all other heterodimers (DQ2, DQ3, and DQ4 by serology) (32–34). Using this approach, Maguire et al. (35) reported that a significant proportion of patients who developed *de novo* HLA-DQ DSAs met one of two criteria: either the recipient was homozygous for one evolutionary group while the donor had alleles from the other group, or the donor had a mismatched HLA-DQ protein containing the DQα05 polypeptide. These observations suggest that evolutionary lineages of HLA-DQ proteins may have clinical relevance, a hypothesis further supported by *in vitro* studies indicating that these differences can influence T cell alloreactivity.

## Complexity of HLA-DQ protein expression and immunogenicity

**Figure 1:**
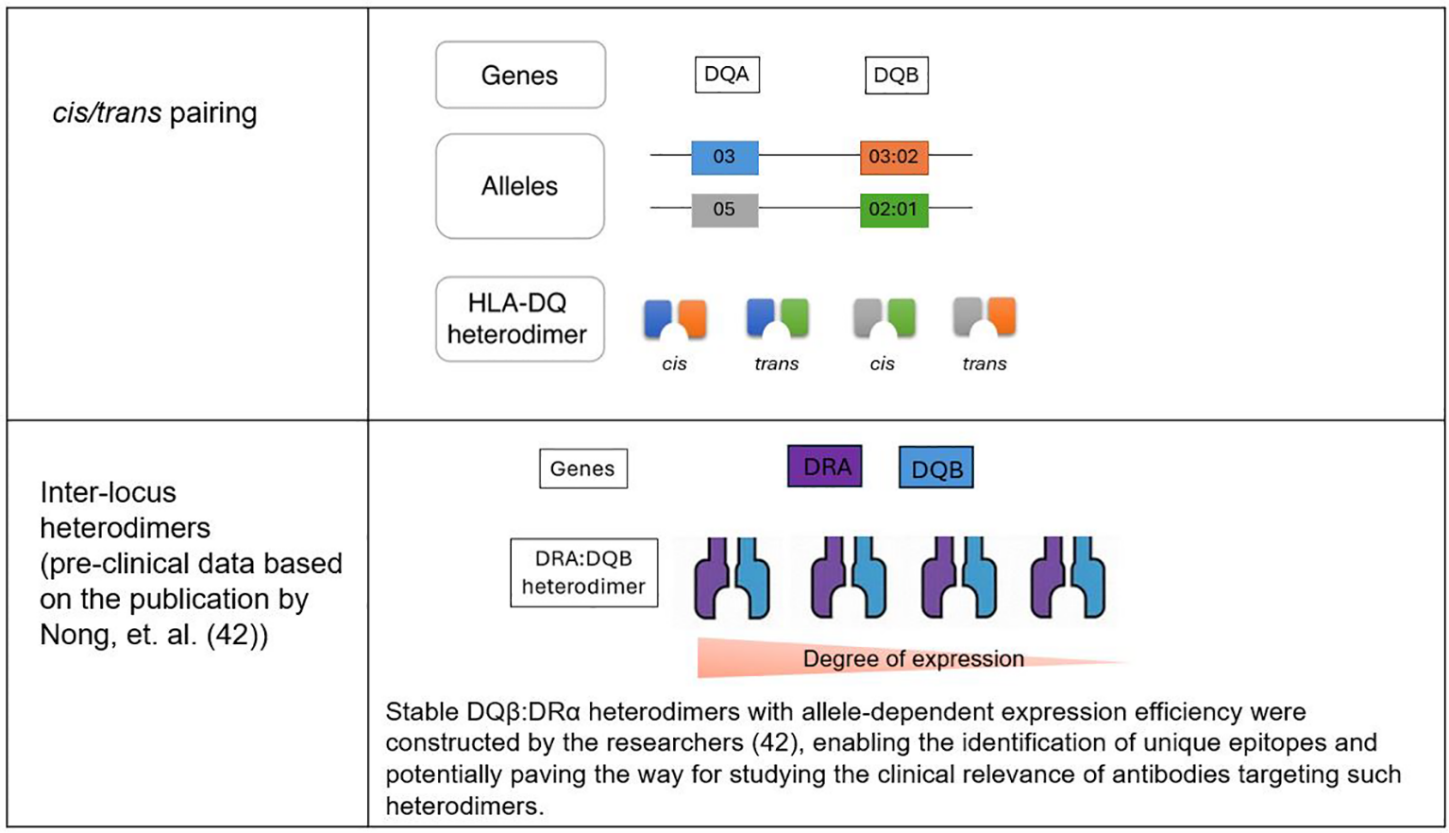
HLA-DQ heterodimers in organ transplantation.

A complicating factor in understanding HLA-DQ mismatches is the combinatorial diversity of HLA-DQ proteins. The HLA-DQB1 gene, which encodes the β chain of the HLA-DQ molecule, can form heterodimers with various α chains (encoded by HLA-DQA1) either in *cis* (from the same chromosome) or *trans* (from opposite chromosome) (36). As a result, up to four different HLA-DQ proteins can be expressed by a single donor, depending on whether they are homozygous or heterozygous for the relevant alleles (36) (**Figure 1**). This diversity poses a challenge for researchers and clinicians, as it is essential to consider every HLA-DQ protein expressed by the donor to fully understand the impact of HLA-DQ disparities in clinical settings.

Moreover, the immunogenicity of HLA-DQ mismatches extends beyond molecular mismatching schemes. For instance, certain amino acid differences in HLA-DQ proteins may be more immunogenic than others. This concept was explored by using pregnancy as a model to study how exposure to fetal HLA antigens influences the development of HLA antibodies in the mother (37, 38). The investigators found that specific amino acid differences were associated with antibody development, while others were not. This result prompted them to propose an immunogenicity score that could complement eplet-based risk assessments (39).

In another recent study (40), HLA-DQ mismatch, along with HLA-DR mismatch, is independently associated with an increased risk of graft failure, rejection, and death in adult living kidney transplant recipients. The study suggests that prioritizing HLA-DQ mismatch over HLA-DR in donor selection could improve transplant outcomes, but additional data will be needed to more conclusively establish this inference.

## Inter-locus heterodimers and HLA-DQ expression

An intriguing aspect of HLA biology that has recently gained attention is the formation of inter-locus heterodimers, where HLA-DRα polypeptides pair with HLA-DQβ polypeptides. Although these inter-locus heterodimers were initially discovered over 30 years ago (41), they have been largely overlooked due to the assumption that they would not occur naturally in normal cells. However, a recent study demonstrated that these heterodimers are indeed stable and can be expressed on the cell surface (42).

HLA class II antigens typically pair within the same isotype, but interisotypic DQβ: DRα heterodimers have been engineered and shown to be stable. Some DQB1 alleles, such as DQβ0601, formed these heterodimers efficiently on cell surfaces, while others, like DQβ0603, exhibited minimal expression. The presence of a DQα chain did not impact the formation of DQβ: DRα heterodimers. Screening with multiplex bead-based assays identified human sera that specifically reacted to unique epitopes on these heterodimers, especially DQβ*0601: DRα. These findings enable further research, raising the possibility that these structures could have clinical implications that have yet to be fully explored (42).

HLA-DQ expression levels in tissues such as the renal vascular endothelium have been reported to be lower than those of HLA-DR proteins, a finding consistent with the low levels of HLA-DQ mRNA observed in kidney biopsies (43). Experimental studies have shown that while HLA-DR expression can increase dramatically in response to inflammatory stimuli such as interferon-gamma (IFNγ), HLA-DQ expression increases much more modestly (44). This difference in expression levels might influence the immunogenicity and pathogenicity of HLA-DQ mismatches, suggesting that factors regulating HLA-DQ expression should, ideally, be taken into consideration when assessing transplant risk.

Additionally, other factors related to HLA-DQ expression, trafficking, and function could significantly influence the development and pathogenicity of HLA-DQ DSAs. For instance, HLA-DQ mRNA is subject to alternative splicing, resulting in different protein products that are expressed on the cell surface (45). Furthermore, crosslinking of HLA-DQ proteins by antibodies can trigger intracellular signaling and cell activation, potentially contributing to transplant dysfunction (44). Interestingly, some HLA-DQ antibodies have been found to specifically recognize HLA-DQ proteins with particular HLA-derived peptides in their binding groove, suggesting a more complex interaction between antibodies and their targets than previously understood (46).

## Potential clinical implications and future research questions

The advent of routine high-resolution two-field HLA typing in many clinical histocompatibility testing laboratories has provided the potential ability to more accurately assess donor-recipient mismatches for HLA-DQ antigens. This enhanced base of information on donor-recipient DQ antigen-based incompatibility offers the prospect of future progress on sorting out the relative influence on clinical outcomes of various biochemical complexities associated to a greater degree with HLA-DQ heterodimers than with HLA-DR or -DP antigens. Recapping the points above, these features include the greater equality in amino acid sequence diversity between alpha and beta chains, the existence of both cis and trans heterodimers on cell surfaces, the occurrence of inter-isotypic heterodimers involving DQ beta chains and DR alpha chains, and alternative splicing of DQ transcripts. **Table 1** highlights some of the key points that we have addressed in this mini review. Below, we present several research questions that could guide exploration in this area and lay the groundwork for future studies.

**Table 1:** Key points discussed in this mini review.

Category	Details
Molecular Mismatching & Algorithms ^a^	• Algorithms: HLA Matchmaker, EMMA, PIRCHE-II, EMS-3D. • Methods: Identify eplets, polymorphic solvent-accessible mismatches, T-cell responses, electrostatic potential differences. • Findings: Eplet-based mismatching better correlates with clinical outcomes than traditional antigen-level mismatching.
Immunogenicity Factors ^b^	• Certain amino acid differences could be more immunogenic. • Pregnancy studies show variable antibody development depending on amino acid differences.
Evolutionary Divergence ^c^	• Grouping HLA-DQ proteins by evolutionary lineages (e.g., DQα01 vs. DQα05). • Divergence linked to T-cell alloreactivity and DSA development.
Inter-Locus Heterodimers ^d^	• Stable pairing of HLA-DRα with HLA-DQβ polypeptides. • Low expression levels of HLA-DQ in tissues compared to HLA-DR. • Linked to immune system recognition and transplant dysfunction.
HLA-DQ Protein Diversity ^e^	• Expression: Up to 4 HLA-DQ proteins due to cis/trans heterodimer formation. • Impact: Combinatorial diversity complicates mismatch assessment.
Expression & Function ^f^	• Influenced by alternative splicing and antibody interactions. • HLA-DQ are less responsive to IFNγ compared to HLA-DR. • Crosslinking triggers intracellular signaling and activation

^a^These are computational tools designed for assessing molecular-level mismatching based on eplets or sequence polymorphisms.

^b^This refers to attributes or metrics that influence or quantify a substance’s ability to elicit an immune response.

^c^This refers to the process by which two or more species evolve distinct traits and characteristics from a common ancestor due to different selective pressures or genetic drift over time.

^d^These are protein complexes formed by subunits encoded by different genetic loci, often contributing to diverse biological functions and inter-molecular interactions.

^e^Protein diversity arises from genetic polymorphism contributing to variations in immune response and susceptibility to autoimmune diseases.

^f^Antibody-induced crosslinking of HLA-DQ molecules can lead to downstream intracellular signaling and immunological activation.

Some unanswered questions for the broader transplant community to consider investigating:

1. Factors drive HLA-DQ immunogenicity:
  - Can reliable methods be developed for gauging the impacts of amino acid substitutions in HLA-DQ molecules on immunogenicity?
  - Are some amino acid differences generally more likely to elicit potent alloantibody responses than others?
  - What are the relative contributions of cis/trans heterodimers and interisotypic HLA-DQ: DR heterodimers to the induction of clinically relevant alloimmune responses?
2. Influence of HLA-DQ evolutionary lineages on outcomes:
  - How do mismatches within and across HLA-DQ evolutionary lineages compare in immunogenicity with respect to humoral and/or cell-mediated immune responses?
3. Role of HLA-DQ expression levels on immunogenicity:
  - To what extent does differential expression of HLA-DQ influence alloantibody development, and how does this variation compare to that exhibited HLA-DR and –DP class II molecules?
  - How can the regulation of HLA-DQ expression be manipulated therapeutically?
4. Molecular mismatch tools as predictors for DSA formation and graft failure:
  - Do any of the molecular mismatching algorithms offer reliably greater predictive value for clinical outcomes, and does any such superiority apply in diverse patient populations?
  - Can a unified approach be developed to assess risk based on eplet scores, immunogenicity scores, and other biophysical properties?
5. Clinical relevance of inter-locus heterodimers:
  - Do antibodies against DQβ: DRα heterodimers contribute to allograft injury, and should their detection be integrated into routine DSA testing protocols?
6. Alternative splicing and HLA-DQ trafficking influence on pathogenicity:
  - What are the functional consequences of alternatively spliced HLA-DQ proteins, and how do they affect antigen presentation and immune activation?
  - Could these pathways be targeted to prevent HLA-DQ antibody formation or reduce graft injury?
7. Optimization of therapies for HLA-DQ specific immune responses:
  - What are the most effective strategies for managing patients with high HLA-DQ molecular mismatch scores and DSA development?
  - Would therapies targeting inflammatory cytokines (e.g., IFNγ) reduce HLA-DQ-associated graft injury?
8. Implications of HLA-DQ specific peptide binding:
  - To what extent do peptides bound to HLA-DQ influence the binding and pathogenicity of DSAs?
  - Could therapeutic modulation of peptide-HLA interactions mitigate antibody-mediated damage?
